# The Joint Effects of Bisphenols and Iodine Exposure on Thyroid during Pregnancy

**DOI:** 10.3390/nu15153422

**Published:** 2023-08-02

**Authors:** Wei Lu, Zhuo Sun, Zhengyuan Wang, Mengying Qu, Zehuan Shi, Qi Song, Liping Shen, Shupeng Mai, Yuan Wang, Xinyu Hong, Jiajie Zang

**Affiliations:** Division of Health Risk Factors Monitoring and Control, Shanghai Municipal Center for Disease Control and Prevention, Shanghai 200336, Chinasongqi@scdc.sh.cn (Q.S.); shenliping0516@163.com (L.S.); wangyuan@scdc.sh.cn (Y.W.);

**Keywords:** iodine, thyroid, bisphenols

## Abstract

The aim of this research was to study the combined effects of bisphenols and iodine exposure on the thyroid gland during pregnancy. We included 162 pregnant women from a cohort established in Shanghai. Urinary concentrations of bisphenol A, bisphenol B(BPB), bisphenol C(BPC), bisphenol F, bisphenol S, and bisphenol AF(BPAF) were examined. Bayesian kernel machine regression (BKMR) and quantile g-computation models were used. The geometric means of BPA, BPB, BPC, BPF, BPS, BPAF, and ΣBPs levels in urine were 3.03, 0.24, 2.66, 0.36, 0.26, 0.72, and 7.55 μg/g creatinine, respectively. We observed a positive trend in the cumulative effects of BPs and iodine on serum triiodothyronine (FT3) and free thyroxine (FT4), as well as a U-shaped dose–response relationship between BPs and the probability of occurrence of thyroperoxidase autoantibody positivity in women with low urinary iodine concentration. In addition, a synergistic effect on the probability of occurrence of thyroid autoantibody positivity was observed between BPF and BPB, as well as between BPC and BPAF in this study. There were adverse health effects on the thyroid after co-exposure to BPs and iodine. Even if pregnant women were exposed to lower levels of BPs, women with iodine deficiency remained vulnerable to thyroid autoimmune disease.

## 1. Introduction

Bisphenols (BPs) are a kind of large-scale man-made materials with similar molecular structure. Bisphenol A is a clear endocrine disruptor, and more and more studies showed that bisphenol A substitutes also have endocrine disrupting effects. There is an increasing trend in women’s exposure to bisphenol A (BPA) and alternatives [1,2]. It was reported that tenBPs were detected in the serum samples of women with total BPs (ΣBPs) levels of 0–144 ng/mL in China [3]; high detectable rates of BPA, bisphenol F (BPF), and bisphenol S (BPS) in the urine samples of pregnant women were reported in Korea [4] and Sweden [5]; the mean urinary concentration of ΣBPs was (23.42 ± 57.83) ng/mL in Indian women [6]. Exposure to chemicals may be more harmful during pregnancy and childhood, which are vulnerable times for susceptibility to chemicals. The health effects of exposure to BPs during pregnancy are a matter of concern.

Due to its critical function in fetal growth, brain development, and metabolism, the thyroid hormone is one of the most important endocrine hormones throughout pregnancy [7]. There has been an increase in studies focusing on BPA and analogues and a deepened understanding of their effects on endocrine disruption. Bisphenol analogues were found to have significant thyroid hormone activity toward the rat pituitary cell line GH3 in vitro [8]. Based on population-based study results, exposure to BPA was related to reduced total thyroxine (TT4) in pregnant women and decreased thyrotropin (TSH) in male neonates [9]. Individuals were, however, constantly exposed to a variety of BPs at the same time, and Bisphenol analogues may interact with each other, producing either additive or antagonistic effects. Therefore, it is essential to carry out studies assessing the overall effect of BPs on thyroid.

Thyroid hormones require iodine as a vital component. Appropriate iodine nutrition state is essential for maintaining the metabolism and function for thyroid. The risk of developing a thyroid disorder may be increased by both iodine excess and deficiency [10]. Iodine nutrition condition during pregnancy may modify the effects of BPs exposure on thyroid hormones. However, little research has focused on the overall effects of BPs on thyroid during pregnancy after considering iodine nutrition condition.

Therefore, we conducted a study to investigate the association between gestational co-exposure to BPs and iodine and thyroid status in women from a pregnancy–birth cohort in Shanghai. Our aim was to explore the relationship between co-exposure to bisphenols-iodine mixtures and thyroid dysfunction during pregnancy.

## 2. Materials and Methods

### 2.1. Study Population and Data Collection

Participants in the study were chosen from a cohort that was created in Shanghai in 2017, as detailed previously [11].

In order to rule out potential influences, such as pre-diagnosed diseases in the kidney, cardiovascular system, endocrine, and other systems, that may affect the iodine metabolism and excretion, subjects with chronic disease conditions were excluded from the study. We also excluded participants whose laboratory results were missing. A total of 162 pregnant women were included in this study, and repeated urine samples (*n* = 604) were collected from the first trimester to the third trimester. The Ethical Committee of the Shanghai Municipal Center for Disease Control and Prevention gave its approval to the study (EC No. 2017/13, approval date: 25 April 2017).

### 2.2. Urine Collection and BPs Laboratory Measurements

Pregnant women provided first spot morning urine samples in polypropylene specimen containers, which were then transported to the lab in ice packs and kept at 80 °C until analysis. The BPs were separated on Waters ACQUITY UPLC BEH phenyl Column (1.7 um 2.1 × 100 mm) and quantified via liquid–liquid extraction coupled with ultra-high performance liquid chromatography tandem mass spectrometry (Therm Fisher, Waltham, MA, USA) [12]. The limits of detection (LODs) for urinary BPA, BPB, BPC, BPF, BPS, and BPAF were 0.40, 0.01, 0.18, 0.02, 0.03, and 0.18 μg/L, respectively.

### 2.3. Assessment of Iodine Nutrition Status

Iodine urinary tests were carried out according to the methods detailed in our published articles [11] and urinary iodine concentrations (UIC) were used to assess maternal iodine status. Iodine status was categorized as follows: low UIC (≤149 μg/L); normal UIC (150–249 μg/L); high UIC (≥250 μg/L) [13].

### 2.4. Thyroid Gland Function Assessment

The women’s venous blood (5 mL) collected in first trimester was centrifuged at 1800× *g* for 10 min. Serum was then separated and kept at 80 °C. Using a Cobas Elecsys 602 (Roche Diagnostics, Basel, Switzerland) system, antibody levels against serum thyrotropin (TSH), thyroid peroxidase, thyroglobulin, and thyrotrophic receptor were assessed. Using an automatic luminescent immune analyzer (Roche Diagnostics, Basel, Switzerland), we were able to determine the serum concentrations of TSH, free triiodothyronine (FT3), free thyroxine (FT4), total tri-iodothyronine (TT3), and total thyroxine (TT4). Thyroid peroxidase antibody (TpoAb) positivity was defined as TpoAb ≥ 34 IU/mL, and thyrotrophic receptor antibody (TrAb) positivity was defined as TrAb ≥ 1.75 IU/L [14].

### 2.5. EDI and Risk Assessment

The estimated daily intake (EDI) (expressed in unit of nmol/kg body weight/day), hazard quotient (HQ), and hazard index (HI) for BPs exposures among pregnant women were calculated according to previous papers [15,16]. Given that FUE (the rate of urinary excretion) of BPB, BPC, BPF, or BPAF after oral exposure in humans has not been evaluated by pharmacokinetic studies, we substituted the value of BPA for those substances.

### 2.6. Covariates

Maternal age, education, and annual family income were recorded using a questionnaire. Face-to-face interviews with the pregnant women were conducted by investigators with specialized training. To calculate the subjects’ body mass index (BMI), the subjects’ height and weight were measured using equipment of standardized brands and models.

### 2.7. Statistical Analysis

By dividing the LOD by the square root of 2, the concentration of bisphenol analogues below the LODs was set, and creatinine adjustment was used to account for variations in urinary volume. Natural log transformed (ln-transformed) values of bisphenol analogues were used to improve normality in the analyses because urinary concentrations of these compounds had a skewed distribution. Spearman’s rank coefficients were used to determine the correlations of each bisphenol and UIC. Given that phenols have short half-lives, we averaged the BPs concentrations of several urine samples taken from study participants to determine the average exposure during pregnancy [17].

A linear regression model was constructed with ln-transformed parameters related to thyroid status as dependent variables and quantiles of specific BPs as predictors (using the lowest quantile as reference), adjusted for pre-pregnancy BMI, household income, maternal education, and maternal age. [exp (β) − 1]100% was used to calculate the percent change in thyroid hormones, where β was the coefficient of the linear regression model. We also used multivariate logistic regression to assess the relationship between each bisphenol with TrAb and TpoAb status by comparing the second, third, and fourth quartiles of each bisphenol to the first quartile.

We used the Bayesian kernel machine regression (BKMR) model, described in previous studies [18], to estimate the joint effects of BPs and iodine co-exposure on thyroid. We fitted the models by running the Markov Chain Monte Carlo sampler with 10,000 iterations. We simultaneously used the quantile g-computation model to further explore and quantify the effects of the mixtures on thyroid in order to produce a more conclusive result. All analyses were carried out using Python (version 3.8) and R (version 4.1.2), with the statistical significance level set at α = 0.05 (two-sided).

## 3. Results

### 3.1. Characteristic of Study Populatio

Total of 162 pregnant women were included in this study. As shown in Table S1, most of the participants were under 30 years old, with high educational level and under normal BMI. The number of women who had one parity time (62.35%) were more than women who had two and above parity times (37.65%).

### 3.2. Exposure Levels of Urinary BPA and Its Alternatives in Study Participants

The exposure levels of urinary BPs in pregnant women are presented in Table 1. BPA, the highest concentrations among all detected bisphenol compounds, was detected in 90.36% of pregnant women. The geometric means of creatinine-adjusted BPA, BPB, BPC, BPF, BPS, BPAF, and ΣBPs concentrations in urine samples were 3.03, 0.24, 2.66, 0.36, 0.26, 0.72, and 7.55 μg/g creatinine, respectively. Levels of individual BP were moderately correlated (correlation coefficient: 0.07–0.65, *p* < 0.001) (Figure S1). Little changes in urinary BPs concentrations were found during different gestational periods in pregnant women (Figure S2).

### 3.3. Risk Assessment of Exposure to BPA and Its Alternatives

The EDI, HQ, and HI for exposure to BPA and its alternatives among pregnant women are summarized in Table S2. The mean values of EDIs for BPA, BPB, BPC, BPF, BPS, BPAF, and ∑BPs were 0.3170, 0.0419, 0.2564, 0.1324, 0.0767, 0.0489, and 0.8734 nmol/kg body weight/day, respectively, which were above the full TDI for BPA defined by the European Food Safety Authority (EFSA) (0.2 ng/kg body weight/day). A comparison of this TDI with the dietary exposure showed that both the mean and the 95th percentile dietary exposures in participants exceeded the TDI by two to three orders of magnitude.

### 3.4. Overall Effects of BPs on Thyroid Health

**Figure 1:**
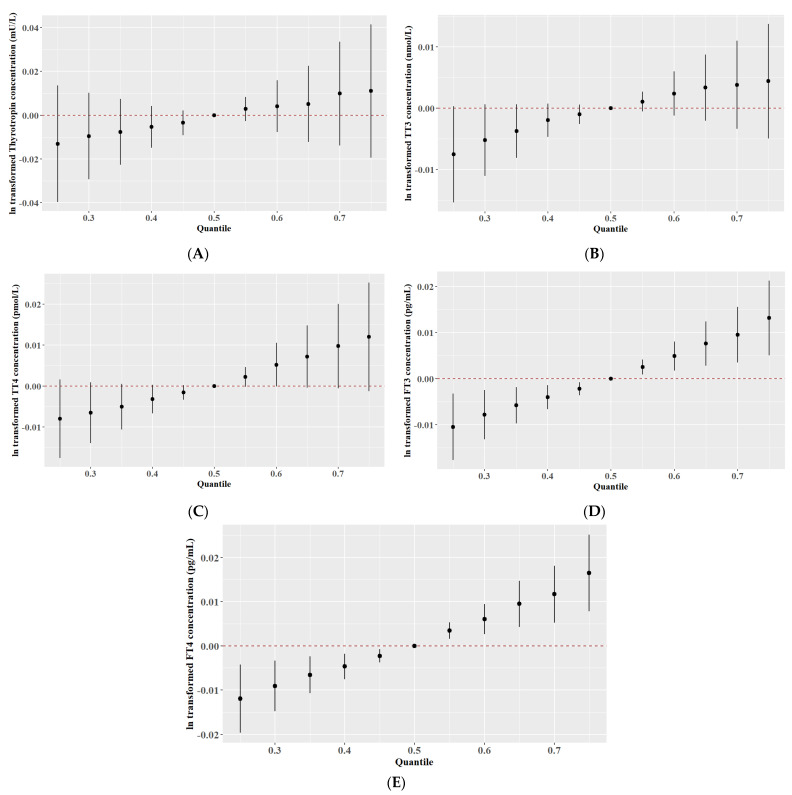
Combined effects of BPs and iodine on thyroid hormones. (**A**–**E**) Overall effects of BPs on thyrotropin, TT3, TT4, FT3, or FT4.

**Figure 2:**
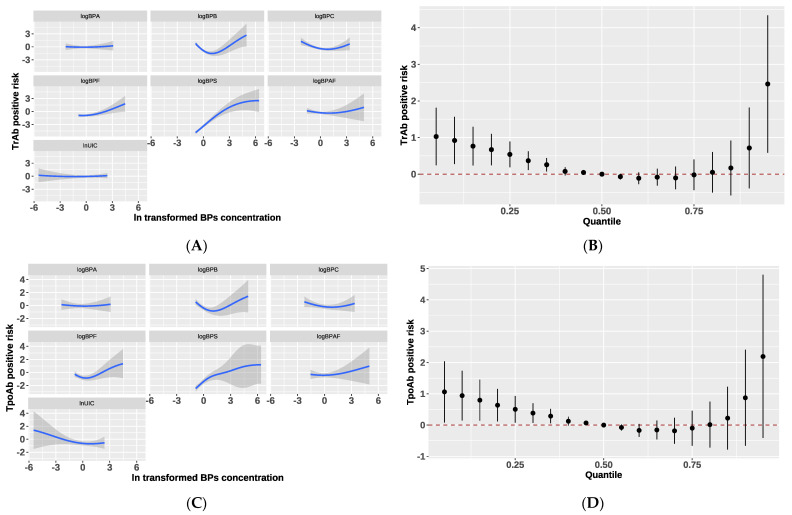
Effects of BPs and iodine exposure on thyroid autoimmune antibodies. (**A**) Univariate exposure–response functions between each ln-transformed BPs (BPA, BPB, BPC, BPF, BPS, or BPAF) or UIC and TrAb-positive risk. (**B**) Combined effects of BPs and iodine on TrAb-positive risk. (**C**) Univariate exposure–response functions between each ln-transformed BPs (BPA, BPB, BPC, BPF, BPS, BPAF) or UIC and TpoAb-positive risk. (**D**) Combined effects of BPs and iodine on TpoAb-positive risk. NOTE: TrAb/TpoAb-positive risk could be understood as the probability of occurrence of TrAb/TpoAb positivity after co-exposure to BPs and iodine. The grey color around bule line in (**A**,**B**) mean the relationship of mixtures with TrAb- or TpoAb-positive risk together with pointwise 95% credible intervals.

We analyzed the estimated change in thyroid hormones when all the BPs were at a certain percentile (from the 10th to 85th percentile) compared with when they were at their median values (50th percentile) using BKMR [18]. A significant positive trend of the cumulative effect of BPs on serum FT3 and FT4 is shown in Figure 1. Increasing levels of the BPs were significantly associated with an increase in serum FT4 and FT3 concentrations. Interestingly, there was a U-shaped dose–response relationship between BPs-iodine mixtures and TrAb- (Figure 2B) or TpoAb-positive risk (Figure 2D) in the probit BKMR model. However, after being stratified by iodine status, the U-shaped dose–response relationship between BPs and TpoAb-positive risk was only observed in the low UIC group (Figure S5(A2)).

### 3.5. Association between Each BPs and Thyroid Health

Firstly, the percent changes (95% CI) in serum thyroid hormones associated with quantiles of BPs was analyzed based on a linear regression model, as shown in Figure S3. After adjusting for potential confounders, BPB concentrations were negatively associated with TT3, FT3, and FT4 (*p* for trend = 0.004, 0.011, and 0.013, respectively), and BPS concentrations were negatively associated with TT3 (*p* for trend = 0.023).

Secondly, BKMR was used to estimate the exposure–response relationship between each ln-transformed bisphenol exposure and thyroid hormones, as shown in Figure S4. There was an inverted U-shaped dose–response relationship between BPAF and TSH; BPS and FT4. We could also observe a positive trend between BPF and serum TT4; BPS and FT3.

Thirdly, we also analyzed the associations of each BPs with TrAb and TpoAb status using logistic regression models in Table S3. Increasing levels of BPS were associated with an increased risk of TrAb or TpoAb positivity (*p* for trend < 0.0001), while increasing levels of BPB were associated with a decreased risk of TrAb and TpoAb positivity (*p* for trend < 0.05). Women in the highest quartile of BPS concentrations had a higher risk of TrAb positivity (OR: 31.09, 95%CI: 7.52, 128.51) and TpoAb positivity (OR: 11.67, 95%CI: 3.19, 42.70).

Finally, we used probit BKMR to analyze the associations of each BPs with TrAb- or TpoAb-positive risk. There was a U-shaped dose–response relationship between BPB, BPC, BPAF and TrAb- or TpoAb-positive risk (Figure 2A), and BPS was positively associated with TrAb- or TpoAb-positive risk (Figure 2C).

Quantile g-computation was used to study the positive or negative weights of each BPs in association with thyroid hormones and the joint association of BPs concentrations on thyroid hormones, as shown in Figure S6. We found a similar pattern to that seem in the BKMR model, indicating that individual BPs influenced thyroid hormones in different directions and BPs were associated with an increase trend in serum FT4 and FT3 concentrations.

### 3.6. Interaction Effect between BPs and Iodine on Thyroid Antibodies

To study the interaction effect of BPs and iodine, we include UIC into the BKMR model, in which all of the other exposures were fixed at the median quantile [19]. There was a synergistic effect between BPF and BPB as well as BPC and BPAF on the risk of TrAb positivity (Figure S7) and TpoAb positivity (Figure S8). The relative risk of TrAb or TpoAb positivity after BPB exposure was increased with increasing quantiles of BPF exposure, and the relative risk of TrAb or TpoAb positivity after BPF exposure was also increased with increasing quantiles of BPB exposure. The same synergistic effects were also seen between BPAF and BPC on the risk of TrAb positivity. Interestingly, the effect of iodine on the risk of TpoAb positivity was increased after BPB and BPS co-exposure. However, the effect of BPB or BPS on the risk of TpoAb positivity was influenced by iodine exposure. The same results were also observed in BPA, BPAF, BPC, and iodine co-exposure.

## 4. Discussion

Pregnant women are generally both exposed to environmental pollutants and nutrients, rather than to a single one. A positive trend of the cumulative effect of six bisphenol mixtures on FT4 and FT3 was shown in this study. There was a U-shaped dose–response relationship between BPs and TpoAbs-positive risk in women with low UIC based on BKMR models. In addition, we found a synergistic effect between BPF and BPB as well as BPC and BPAF in this study.

Humans are exposed to BPs through dietary and nondietary sources because it is ubiquitous in the environment. BPA was detected in 90.36% of samples in the study, and it was the most ubiquitous bisphenol among pregnant women. The detection rates of other interested BPs were all above 70%, higher than those reported in Czech [20], Shanghai [21], and the Netherlands [22]. The median concentrations of urinary BPA (unadjusted level = 1.30 μg/L; creatinine-adjusted level = 2.64 μg/g Cr) in this research were comparable to those reported in Wuhan, China (1.18 μg/L) [23] and France (2.00 μg/L) [24], but they were higher than those reported in a study in Shanghai, China (1.13 μg/g Cr) [21]. The creatinine-adjusted geometrical mean urinary concentrations of BPF (0.36 μg/g Cr) were lower than those reported in a study in Shanghai, China (0.53 μg/g Cr) [25], but they were higher than those reported in a study in the Netherlands (0.2 μg/g Cr) [24] and Spain (0.04 μg/g Cr) [26]; BPS (0.26 μg/g Cr) was higher than that reported in a study in Shanghai, China (0.02 μg/g Cr) [25] and the Netherlands (0.20 μg/g Cr) [24]. The median concentrations of BPAF (0.61 μg/g Cr) were lower than those reported in Shanghai, China (0.09 μg/g Cr) [21]. This study found high levels of BPC, which were greater than those found in Switzerland(0.25 μg/g Cr) [27] and Poland (0.016 μg/L) [28]. Different population exposure levels to BPA and its substitutes may be caused by variations in demography, eating habits, and lifestyle.

The EDI can reflect relatively comprehensive exposure levels from a variety of pathways in the body. In our study, the EDI of BPs was above the full TDI defined by EFSA in 2023 (0.2 ng/kg body weight/day) [29]. According to a urinary biomarker, the EDI of BPA among pregnant women worldwide is 0.1841 nmol/kg body weight/day, which is lower than the EDI found in our study (0.3170 nmol/kg body weight/day).

There was an adverse health effects on thyroid after BPs and iodine co-exposure. Increasing levels of BPs and iodine mixture were significantly associated with an increase in serum FT4 and FT3 concentrations. To our knowledge, little study focused on the effect of BPs and iodine co-exposure on thyroid. A study in China reported that high levels of BPB and BPF exposure could increase FT4 levels in the second trimester regardless of iodine status [30]. The common cause of high thyroid hormones in serum was Grave’s disease due to thyroid stimulation by TrAbs [31]. We found a U-shaped dose–response relationship between BPs and TpoAbs-positive risk in women with low UIC, meaning that women with iodine deficiency were still vulnerable to thyroid autoimmune disease when exposure to lower levels of BPs.

According to the results of BKMR and quantile g-computation, individual BPs affected thyroid hormones in different directions. BPB or BPC exposure increased TT3 levels, but exposure to BPS decreased TT3 levels in this study. To our knowledge, the effects of BPA alternatives on thyroid hormones are still contradictory. In zebrafish, a significant decline in thyroid hormone thyroxine (T4) content was observed after exposure to BPB and BPS [32,33]. Lower FT4 levels were associated with higher urinary BPF concentrations in girls [34], while higher BPB levels could lead to higher FT4 levels in pregnant women in the second trimester [30]. According to a cohort study from Puerto Rico, pregnant women exposed to BPF had higher FT4 levels [35]. In addition, a positive trend of the cumulative effect of six bisphenol mixtures on serum thyrotropin, TT4, and FT4 was shown in this study. Since this study is the first to calculate the combined effects of six BPs and iodine on thyroid hormones, no comparative studies exist. A study on the joint toxicity of BPA, BPB, BPF, BPS, and TBBPA in pregnant women reported that an inverted U-shaped dose–response relationship was found on FT3 [30].

According to studies using in vitro and in vivo models, there are both adverse and beneficial effects of co-exposure to BPA and other natural chemicals or environmental stressors [36]. In an in vitro study, there was a synergistic effect between BPA and BPF or BPS [37]. In an epidemiological study, maternal co-exposure to nonylphenol and BPA could increase 8-hydroxy-2′-deoxyguanosine, a biomarker of oxidative stress [38]. There was a synergistic effect between BPF with BPB as well as BPC and BPAF in this study. Another interesting finding in this study was that the effect of iodine could be influenced by BPs, but iodine did not affect the outcome caused by BPs exposure. Higher weights of BPs and posterior inclusion probabilities (PIPs) in the model compared to iodine may explain the results (Figure S15). More research is required to determine how BPs and iodine interact to affect health outcomes.

Unknown biological mechanisms underlie the negative effects of mixed exposure to BPs on the thyroid. One study in Spain found some evidence of the effect of modification of deiodinase (DIO) gene polymorphisms with stronger negative associations between methylparaben, propylparaben, butylparaben, and TT3 as well as BPA and FT4 for DIO1 in women [39]. In zebrafish larvae, transthyretin expression increased in response to BPAF, indicating that both thyroid hormone transport and metabolism were disturbed. BPAF also decreased levels of thyroid hormones and deiodinases [40]. Thyroid hormone receptors (TR) expression was significantly altered in vitro by BPA, BPB, and BPAF; only BPAF significantly decreased TR expression, and an inverted U-shaped dose effect and changes in reactive oxygen species levels were observed after BPs exposure [41], indicating that oxidative stress and hormone receptor disorder may refer to the mode of action. Further research is needed to fully understand the biological mechanisms because different BPs interact with one another, and the combined effects of compounds are complicated.

The current study has several strengths. Firstly, we employed the BKMR statistical method and Quantile g-computation model to assess the joint effects of BPs exposures on thyroid after considering the iodine status. Secondly, repeated spot urine samples were collected to improve the exposure assessment of BPs. Thirdly, the interaction effect between BPs and iodine on thyroid antibodies was also analyzed in our study. However, there are several limitations. In the first place, the sample size in our study was a shortcoming. Secondly, only one serum sample was analyzed to determine each participant’s thyroid status. Thirdly, current cross-sectional study has an inherent limitation of the reverse causation.

## 5. Conclusions

There was an adverse health effect on thyroid after BPs and iodine co-exposure. Even if pregnant women were exposed to lower levels of BPs, women with iodine deficiency were still vulnerable to thyroid autoimmune disease.

## Figures and Tables

**Table 1:** Concentrations of BPA and its substitutes in pregnant women’s urine (*n* = 604).

BPs	Percent Detection	Mean (95% CI)	Selected Percentiles					Geometric Mean (95% CI)
			10%	25%	50%	75%	90%	
Unadjusted (ng/mL)								
BPA	90.36	2.35 (1.88, 2.82)	0.63	0.81	1.30	2.08	3.65	1.44 (1.34, 1.53)
BPB	77.29	0.32 (0.26, 0.37)	0.02	0.04	0.10	0.36	0.85	0.11 (0.10, 0.13)
BPC	86.00	2.08 (1.86, 2.29)	0.34	0.62	1.24	2.42	4.86	1.27 (1.18, 1.39)
BPF	79.01	0.77 (0.62, 0.92)	0.03	0.05	0.13	0.49	2.23	0.19 (0.16, 0.21)
BPS	84.76	0.31 (0.24, 0.39)	0.04	0.06	0.11	0.26	0.65	0.14 (0.13, 0.15)
BPAF	79.94	0.28 (0.27, 0.29)	0.20	0.22	0.26	0.33	0.40	0.27 (0.26, 0.28)
ΣBPs	-	5.29 (4.75, 5.83)	1.15	1.94	3.56	6.37	10.78	3.41 (3.15, 3.68)
Creatinine-adjusted (μg/g creatinine)								
BPA	-	8.20 (3.52, 12.88)	0.99	1.52	2.64	5.19	10.42	3.03 (2.78, 3.30)
BPB	-	0.73 (0.50, 0.97)	0.04	0.07	0.22	0.79	1.69	0.24 (0.21, 0.27)
BPC	-	6.52 (2.75, 10.28)	0.74	1.24	2.67	5.06	10.08	2.66 (2.43, 2.92)
BPF	-	1.57 (1.18, 1.97)	0.06	0.12	0.25	1.01	4.64	0.36 (0.31, 0.42)
BPS	-	0.76 (0.48, 1.04)	0.06	0.11	0.24	0.55	1.32	0.26 (0.24, 0.29)
BPAF	-	1.52 (0.91, 2.12)	0.22	0.37	0.61	1.25	2.72	0.72 (0.65, 0.78)
ΣBPs	-	16.72 (8.28, 25.17)	2.53	4.14	7.88	14.37	25.17	7.55 (6.94, 8.21)

## Data Availability

The data presented in this study are available on request from the corresponding author. The data are not publicly available due to policy.

## Supplementary Materials

The following supporting information can be downloaded at: https://www.mdpi.com/article/10.3390/nu15153422/s1. Table S1. Exposure level of bisphenols in study population based on different characteristic; Table S2 Estimated daily intakes (EDI, nmol/kg body weight/day) and risk assessment for exposure to BPA and its alternatives among pregnant women; Table S3. Adjusted odds ratios (95% CI) of thyroid antibodies by quartiles of BPs (N = 162); Figure S1 Pearson correlation coefficients between pairs of BPs concentrations and urinary iodine concentrations; Figure S2. Changes of urinary BPs concentrations according to gestational age in pregnant women; Figure S3. Adjusted percent changes (95% CI) in serum thyroid hormones associated with quantiles of BPs in linear regression model; Figure S4. Univariate exposure–response functions between each ln-transformed bisphenol (BPA, BPB, BPC, BPF, BPS, BPAF) or UIC and thyroid hormones. (A–E) Univariate exposure–response functions between each bisphenol exposure or UIC and serum thyrotropin, TT3, TT4, FT3 or FT4; Figure S5. Overall effects of BPs exposure on thyroid autoimmune antibodes after being stratified by iodine status. Combined effects of BPs mixtures on TrAb positive risk (A1) and TpoAb positive risk (A2) in women with low UIC. Combined effects of BPs mixture on TrAb positive risk (B1) and TpoAb positive risk (B2) in women with normal UIC. Combined effects of BPs mixture on TrAb positive risk (C1) and TpoAb positive risk (C2) in women with high UIC; Figure S6. Joint association of bisphenol mixture on thyroid hormones estimated using the Quantile-g computation method. (A1–E1) The weighs represent the proportion of positive or negative components of each BPs contributing to TSH, T3, T4, FT3 and FT4. (A2–E2) The overall joint association are interpreted as the changes of thyroid hormones level for each quantile increase in the mixture index; Figure S7. Interaction between pairs of exposures on TrAb positive risk; Figure S8. Interaction between pairs of exposures on TpoAb positive risk; Figure S9. Univariate exposure–response functions and 95% credible intervals for each ln-transformed bisphenol (BPA, BPB, BPC, BPF, BPS, BPAF) and thyroid hormones in women with normal UIC. (A–E) Univariate exposure–response functions between each bisphenol exposure and serum thyrotropin, TT3, TT4, FT3 or FT4; Figure S10. Combined effects of BPs mixture on thyroid hormones in women with normal UIC. (A–E) Overall effects of BPs mixture on thyrotropin, TT3, TT4, FT3 or FT4; Figure S11. Univariate exposure–response functions and 95% credible intervals for each ln-transformed bisphenol (BPA, BPB, BPC, BPF, BPS, BPAF) and thyroid hormones in women with low UIC. (A–E) Univariate exposure–response functions between each bisphenol exposure and serum thyrotropin, TT3, TT4, FT3 or FT4; Figure S12. Combined effects of BPs mixture on thyroid hormones in women with low UIC. (A–E) Overall effects of BPs mixture on thyrotropin, TT3, TT4, FT3 or FT4; Figure S13. Univariate exposure–response functions and 95% credible intervals for each ln-transformed bisphenol (BPA, BPB, BPC, BPF, BPS, BPAF) and thyroid hormones in women with high UIC. (A–E) Univariate exposure–response functions between each bisphenol exposure and serum thyrotropin, TT3, TT4, FT3 or FT4; Figure S14. Combined effects of BPs mixture on thyroid hormones in women with high UIC. (A–E) Overall effects of BPs mixture on thyrotropin, TT3, TT4, FT3 or FT4; Figure S15. The posterior inclusion probabilities (PIPs) of BPs or iodine, which provide a measure of variable importance for each BP or iodine. (A) PIPs of TrAb positive risk (B) PIPs of TpoAb positive risk.
