# Supplementary material for: The Joint Effects of Bisphenols and Iodine Exposure on Thyroid during Pregnancy
Source: Nutrients. 2023 Aug 2;15(15):3422. doi: 10.3390/nu15153422 (PMC10421451; doi:10.3390/nu15153422)
Supplement: Supplementary file 1 [file nutrients-15-03422-s001.zip › nutrients-2497633-supplementary.pdf]

Table S1 Exposure level of bisphenols in study population based on different characteristic

Variable	N(%)[a]	BPA	BPB	BPC	BPF	BPS	BPAF	ΣBPs
Pooled	162	3.51（1.03-15.45）	0.23（0.01-2.46）	3.08（0.54-11.83）	0.48（0.04-7.82）	0.34（0.05-2.81）	0.69（0.09-3.30）	10.99（3.32-33.36）
**Maternal age(years)**								
< 30	96(59.26)	3.96(1.07-16.98)	0.23(0.01-2.99)	3.41(0.61-12.33)	0.55(0.05-9.31)	0.35(0.04-3.80)	0.73(0.10-4.20)	12.36（3.69-40.11）
30-35	50(30.86)	3.05(1.02-11.27)	0.18(0.01-1.33)	2.36(0.17-10.28)	0.27(0.01-3.79)	0.31(0.07-1.97)	0.64(0.20-1.60)	8.65（2.89-24.23）
≥ 35	16(9.88)	2.18(1.03-5.61)	0.38(0.06-6.63)	3.15(1.06-9.72)	0.91(0.08-5.49)	0.34(0.04-1.48)	0.55(0.09-2.04)	9.37（3.77-25.94）
p-value[b]	-	**0.030**	0.743	0.287	0.651	0.729	0.317	0.074
p-value[c]	-	0.237	0.886	0.578	0.754	0.688	0.893	0.323
**Annual household income (1,0000 RMB)**								
< 10	31(19.14)	3.59（0.85-101.39）	0.19（0.01-13.46）	3.76（0.61-384.02）	0.59（0.04-24.4）	0.37（0.04-13.37）	0.68（0.09-13.34）	12.89（3.13-530.22）
10-20	59(36.42)	3.53（1.21-14.30）	0.25（0.02-2.49）	2.77（0.52-9.93）	0.41（0.04-4.48）	0.32（0.05-2.53）	0.63（0.10-2.55）	10.30（3.45-26.82）
20-35	59(36.42)	3.43（0.92-15.87）	0.20（0.01-1.41）	3.16（0.61-12.69）	0.52（0.05-10.42）	0.36（0.04-2.63）	0.73（0.16-3.35）	11.08（3.90-30.24）
≥ 35	13(8.02)	3.65（1.47-9.31）	0.28（0.02-2.92）	3.47（1.06-21.77）	0.53（0.06-4.59）	0.34（0.12-1.10）	0.90（0.46-3.71）	11.24（4.36-38.85）
p-value[b]	-	0.937	0.846	0.916	0.947	1.000	0.432	0.750
**Educational level（years）**								
≤ 9	35(21.61)	4.08（1.19-16.36）	0.31（0.02-4.48）	4.20（0.98-16.90）	0.53（0.04-6.40）	0.29（0.05-7.43）	0.80（0.09-5.32）	13.26（4.40-39.20）
10-15	10(6.17)	2.88（1.16-9.95）	0.18（0.01-1.46）	2.41（0.50-9.12）	0.48（0.05-9.00）	0.29（0.03-2.16）	0.57（0.09-2.33）	8.79（3.08-27.15）
≥ 16	67(41.36)	3.87（1.00-17.16）	0.23（0.01-1.89）	3.32（0.58-11.95）	0.45（0.03-5.41）	0.44（0.06-4.16）	0.74（0.23-2.75）	12.13（3.60-43.07）
p-value[b]	-	0.934	0.486	0.491	0.650	0.0969	0.972	0.934
p-value[c]	-	0.961	0.303	0.535	0.677	0.114	0.910	0.969
**Pre-pregnancy BMI (kg/m2)**								
<18.5	31(19.14)	3.03（1.46-7.86）	0.29（0.04-1.92）	3.06（0.59-11.44）	0.41（0.03-6.34）	0.34（0.04-3.44）	0.67（0.09-3.3.）	10.10（3.66-36.78）
18.5-24	103(63.58)	3.54（0.96-15.41）	0.20（0.01-2.45）	2.88（0.24-11.97）	0.88（0.05-11.27）	0.48（0.10-1.55）	0.69（0.14-3.56）	11.03（3.51-29.59）
24-28	25(15.42)	4.34（1.48-16.51）	0.28（0.02-3.10）	6.48（4.18-10.63）	0.09（0.06-0.14）	0.10（0.05-0.28）	0.26（0.20-0.34）	12.19（3.28-25.60）
≥28	2(1.23)	1.41（1.40-1.43）	0.10（0.04-0.34）	0.61（0.07-11.41）	0.29（0.05-1.97）	0.79（0.38-2.45）	0.22（.0.02-1.24）	8.67（5.96-13.13）

p-value[b]	-	0.511	0.695	0.888	0.932	0.495	0.390	0.624
p-value[c]	-	0.512	0.787	0.790	0.104	0.530	0.980	0.629
**Parity (times)**								
1	101(62.35)	3.38（0.72-15.40）	0.24（0.01-2.45）	2.84（0.51-11.82）	0.43（0.04-6.34）	0.35（0.04-4.51）	0.70（0.09-3.30）	10.69（3.11-29.59）
≥2	61(37.65)	3.71（1.35-15.17）	0.21（0.01-1.66）	3.44（0.61-11.22）	0.56（0.04-8.35）	0.33（0.05-1.82）	0.66（0.14-2.65）	11.44（4.38-34.58）
p-value[b]	-	0.600	0.596	0.325	0.321	0.750	0.738	0.670

[a] Every participant contain 4 collection time point, and BPs concentration were calculated as mean

[b] *p*-values for trend based on bivariate regression models.

[c] *p*-values for continuous variables based on bivariate regression models.

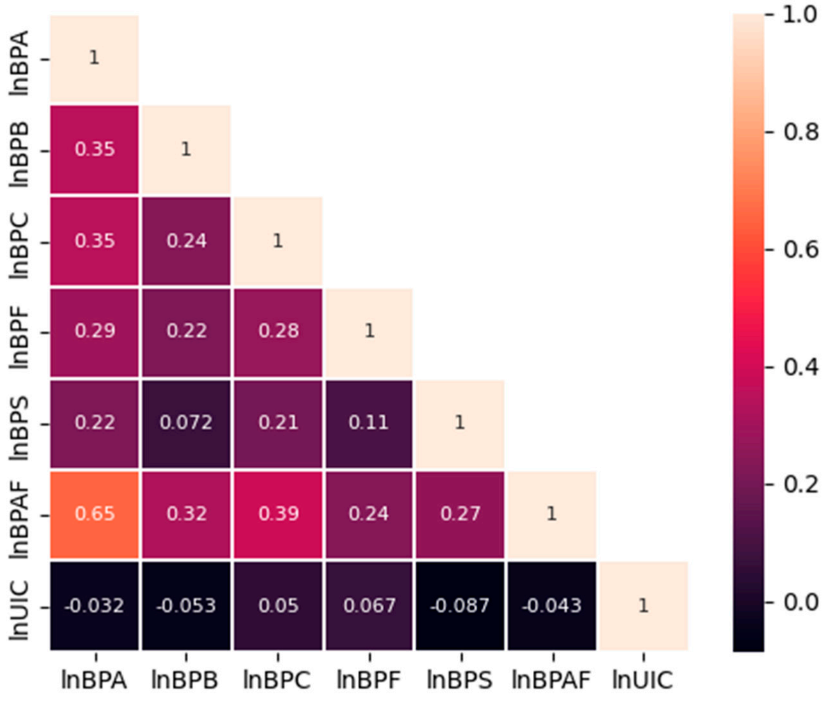

Figure S1 Pearson correlation coefficients between pairs of BPs concentrations and urinary iodine concentrations

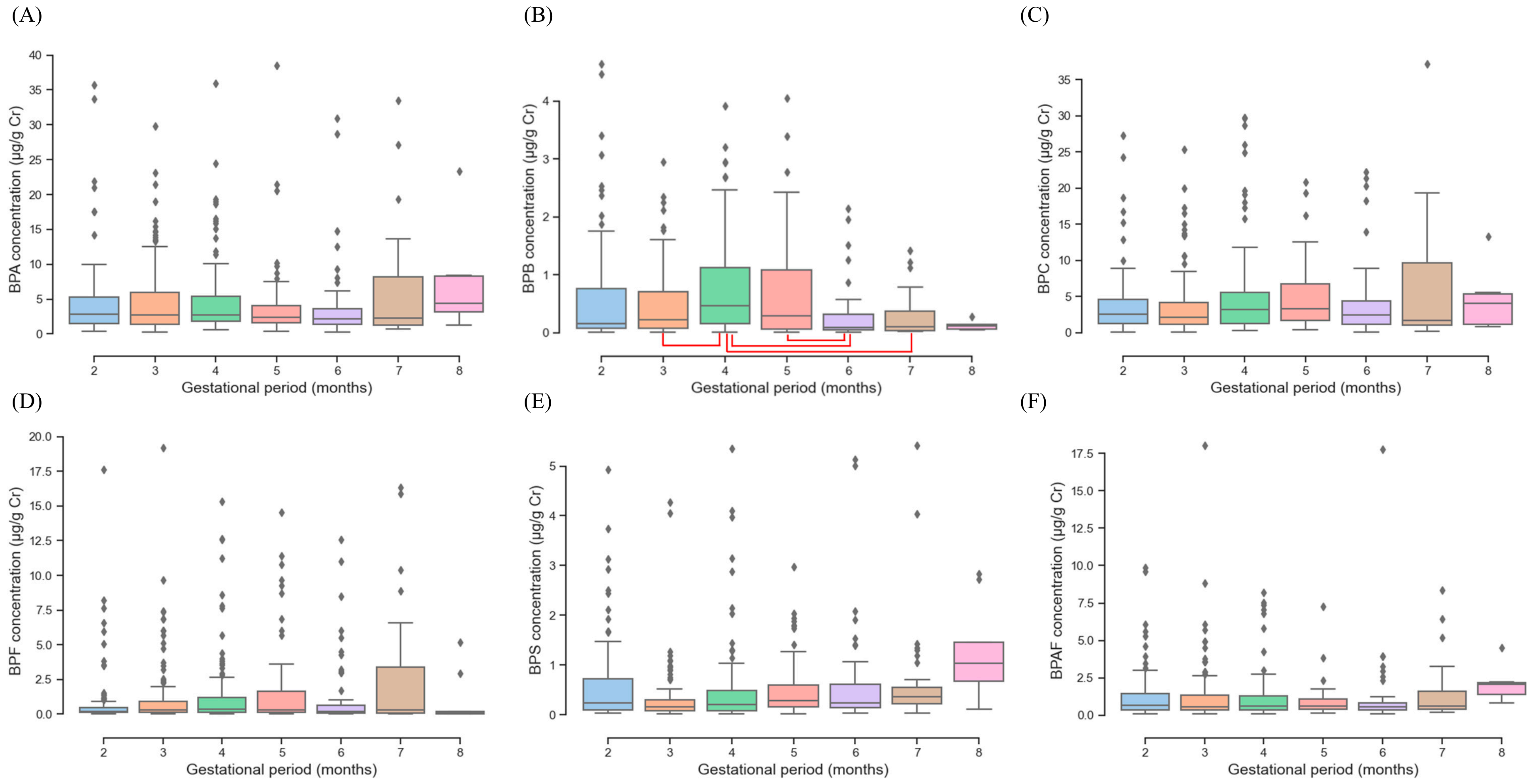

Figure S2. Changes of urinary BPs concentrations according to gestational age in pregnant women

Table.S2 Estimated daily intakes (EDI, nmol/kg body weight/day) and risk assessment for exposure to BPA and its alternatives among pregnant women.

Bisphenols	Average	Selected percentiles					N(%)
		25th	50th	75th	95th	100th	
EDI							EDI>TDI
BPA	0.3170	0.1407	0.2296	0.3937	0.9306	1.3846	
BPB	0.0419	0.0057	0.0191	0.0419	0.1412	0.7501	148(91.36)
BPC	0.2564	0.0997	0.1765	0.3262	0.7150	2.2683	158(97.50)
BPF	0.1324	0.0111	0.0341	0.1459	0.4755	1.7492	128(79.01)
BPS	0.0767	0.0121	0.0301	0.0724	0.2621	1.7860	137(84.57)
BPAF	0.0489	0.0192	0.0325	0.0568	0.1392	0.3762	147(90.74)
ΣBPs	0.8734	0.4373	0.7189	1.1511	2.0332	3.9572	160(98.80)
HQ							HQ>1
BPA	361.89	160.63	262.07	449.39	1062.38	1580.59	162(100.00)
BPB	47.86	6.47	21.79	47.78	161.24	856.23	148(91.36)
BPC	292.73	113.77	201.49	372.43	816.16	2589.36	162(100.00)
BPF	151.14	12.65	38.98	166.54	542.76	1996.85	159(98.15)
BPS	87.58	13.81	34.38	82.63	299.21	2038.83	162(100.00)
BPAF	55.86	21.88	37.08	64.83	158.88	429.44	162(100.00)
HI	997.06	499.20	820.68	1314.09	2321.03	4517.36	-

TDI: tolerable daily intakes; HQ: hazard quotient, and HI: hazard index.

Table S3. Adjusted odds ratios (95% CI) of thyroid antibodies by quartiles of BPs (N=162)

**BPs**	**Case/Non-case**	**TrAb positive odds ratio (95% CI)**	**Case/Non-case**	**TpoAb positive odds ratio (95% CI)**
lnBPA				
Quartile 1	17/24	Ref	14/27	Ref
Quartile 2	9/31	0.47(0.17,1.27)	7/33	0.38(0.13,1.11)
Quartile 3	14/26	0.94(0.37,2.40)	14/26	1.09(0.42,2.84)
Quartile 4	18/23	1.21(0.50,2.99)	15/26	1.06(0.42,2.69)
P for trend		0.2295		0.4225
lnBPB				
Quartile 1	27/14	Ref	22/21	Ref
Quartile 2	12/28	**0.19(0.07,0.50)**	12/28	**0.43(0.17,1.10)**
Quartile 3	8/32	**0.12(0.04,0.35)**	8/32	**0.24(0.09,0.67)**
Quartile 4	11/30	**0.20(0.75,0.52)**	10/31	**0.34(0.13,0.87)**
P for trend		**0.0025**		**0.0374**
lnBPC				
Quartile 1	21/20	Ref	16/25	Ref
Quartile 2	9/31	**0.27(0.10,0.71)**	9/31	0.44(0.16,1.17)
Quartile 3	14/26	0.47(0.19,1.19)	13/27	0.66(0.26,1.69)
Quartile 4	14/27	0.53(0.21,1.33)	12/29	0.60(0.23,1.52)
P for trend		0.1334		0.4018
lnBPF				
Quartile 1	**19/22**	Ref	15/26	Ref
Quartile 2	10/30	0.42(0.16,1.10)	10/30	0.61(0.23,1.60)
Quartile 3	8/32	**0.30(0.11,0.84)**	7/33	**0.34(0.12,0.99)**
Quartile 4	21/20	1.25(0.52,3.03)	18/23	1.45(0.59,3.57)
P for trend		0.2338		0.2518
lnBPS				
Quartile 1	5/36	Ref	5/36	Ref
Quartile 2	7/33	1.38(0.39,4.90)	8/32	1.51(0.43,5.32)
Quartile 3	**18/22**	**8.08(2.41,27.03)**	**17/32**	**8.72(2.54,29.96)**
Quartile 4	**28/13**	**21.70(6.25,75.30)**	**20/21**	**10.58(3.11,36.03)**
P for trend		**0.0001**		**0.0001**
lnBPAF				
Quartile 1	18/23	Ref	14/27	Ref
Quartile 2	10/30	0.47(0.18,1.23)	9/31	0.56(0.20,1.53)
Quartile 3	17/23	0.99(0.40,2.43)	13/27	1.00(0.39,2.56)
Quartile 4	13/28	0.66(0.26,1.67)	14/27	1.00(0.39,2.56)
P for trend		0.6583		0.7271

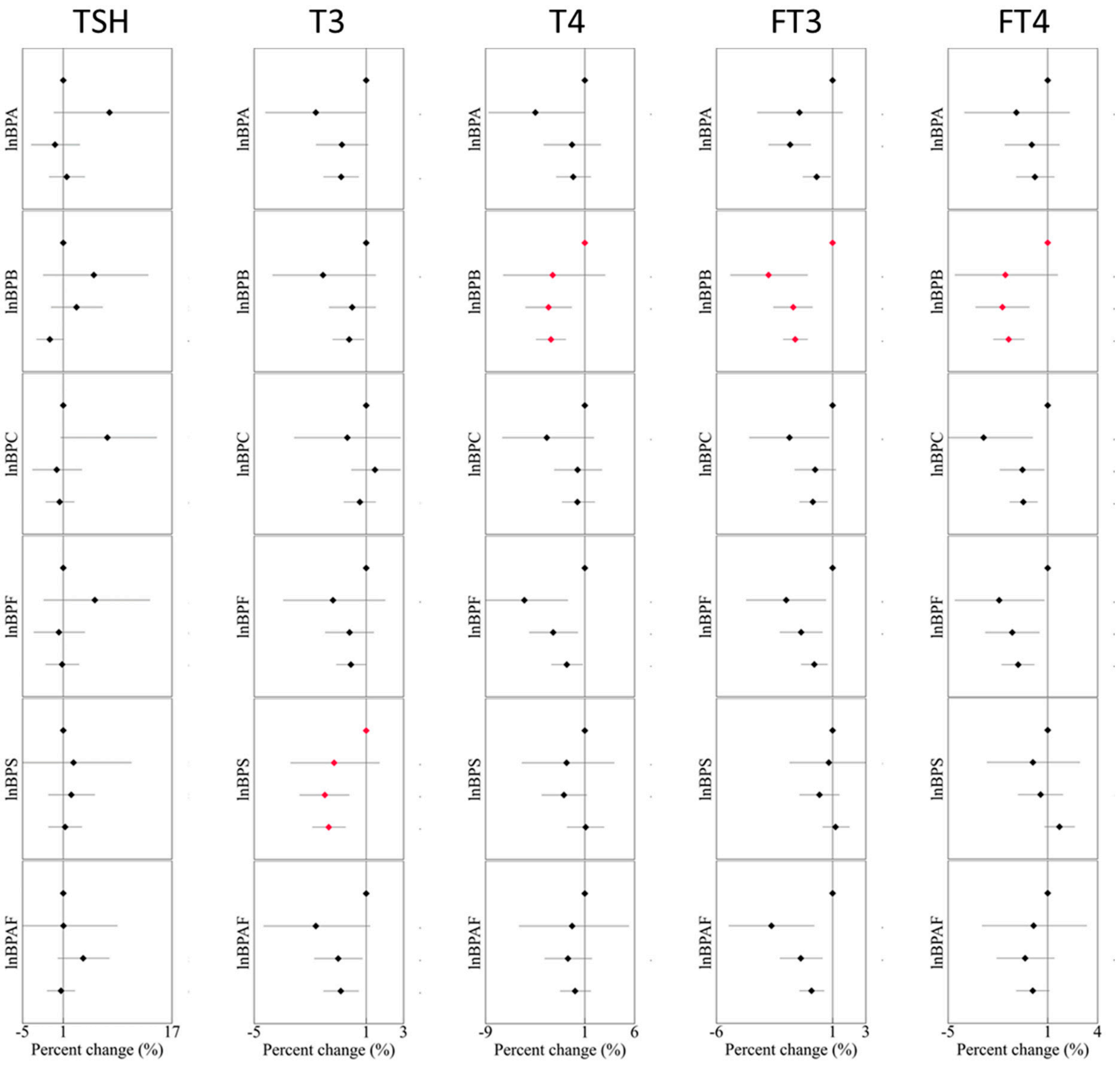

Figure S3. Adjusted percent changes (95% CI) in serum thyroid hormones associated with quantiles of BPs in linear regression model. The dots and horizontal bars represent the percent changes and corresponding 95% CIs, respectively. Solid dots in red mean positive associations with statistical significance in *P* for trend test based on linear regression model ($P < 0.05$). linear regression model was constructed with thyroid hormone (ln-transformed) as dependent variable and quantiles of specific bisphonel as predictors (using the lowest quantile as reference), adjusted for maternal age, maternal education, household income, and pre-pregnancy BMI. Percent change in thyroid hormones was calculated as [exp (β)-1]×100%, where β was the coefficient of the linear regression model.

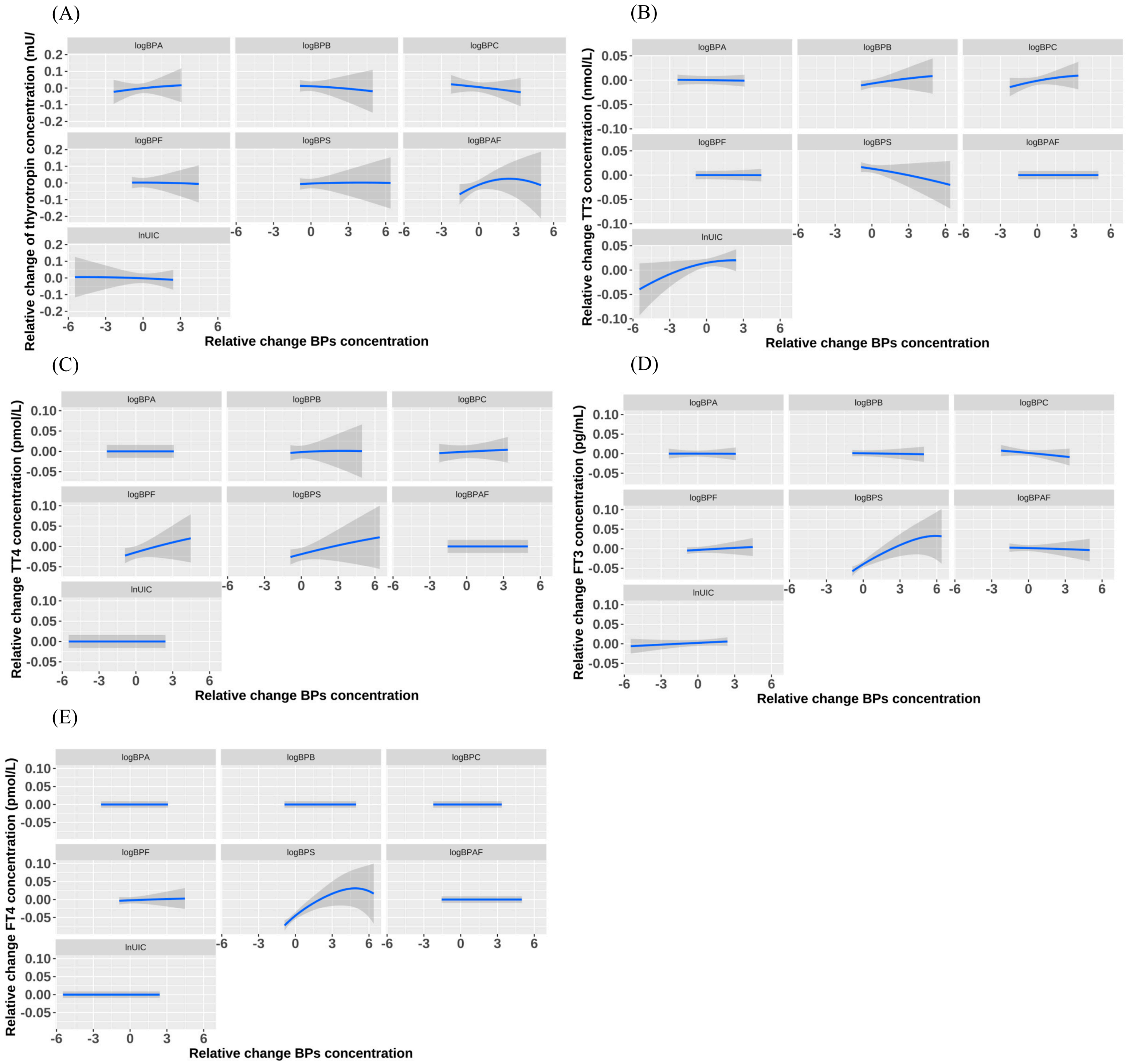

Figure S4. Univariate exposure–response functions between each ln-transformed bisphenol (BPA, BPB, BPC, BPF, BPS, BPAF) or UIC and thyroid hormones. (A) (B) (C) (D) (E) Univariate exposure–response functions between each bisphenol exposure or UIC and serum thyrotropin, TT3, TT4, FT3 or FT4.

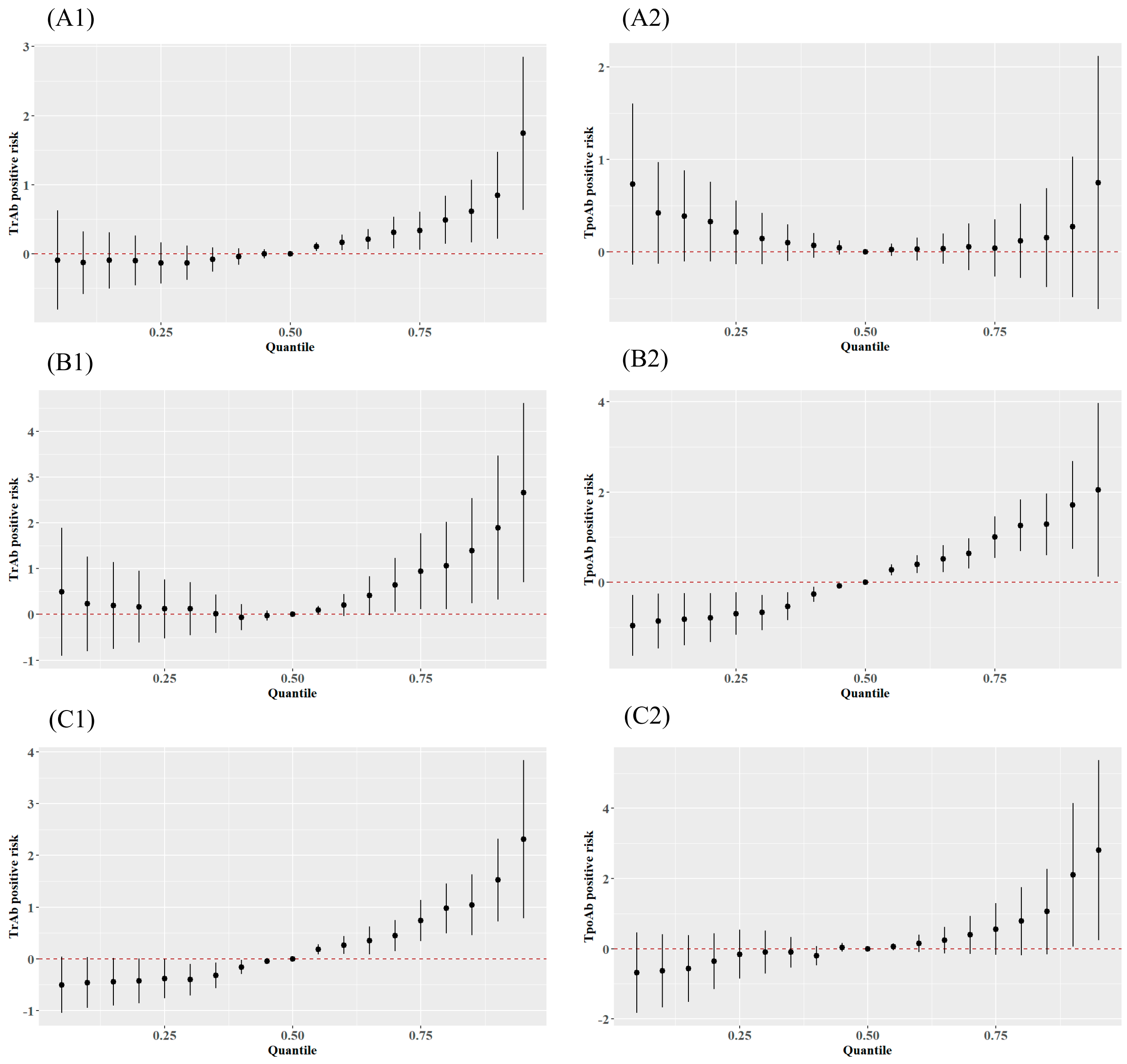

Figure S5. Overall effects of BPs exposure on thyroid autoimmune antibodes after being stratified by iodine status. Combined effects of BPs mixtures on TrAb positive risk (A1) and TpoAb positive risk (A2) in women with low UIC. Combined effects of BPs mixture on TrAb positive risk (B1) and TpoAb positive risk (B2) in women with normal UIC. Combined effects of BPs mixture on TrAb positive risk (C1) and TpoAb positive risk (C2) in women with high UIC.

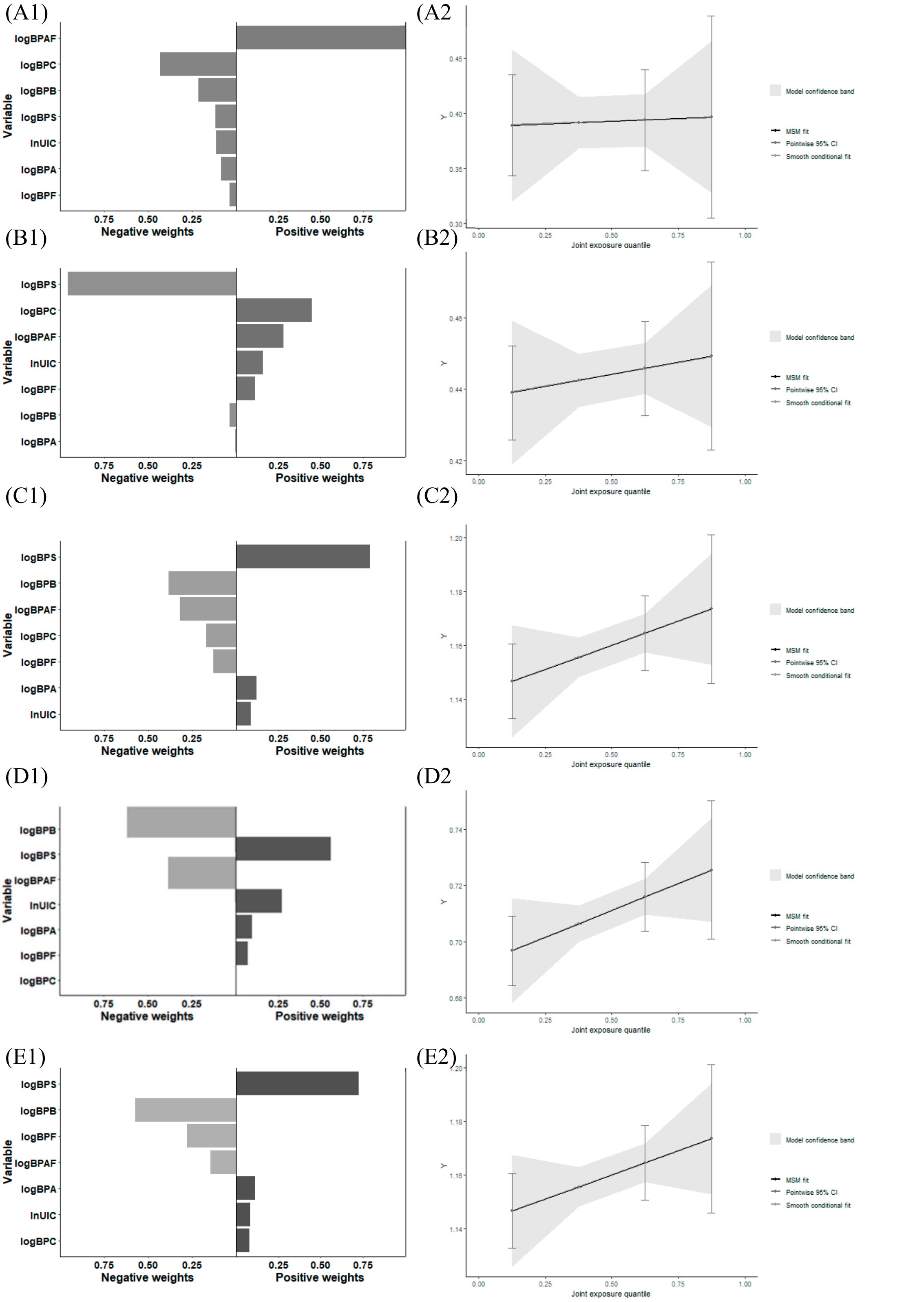

Figure S6. Joint association of bisphenol mixture on thyroid hormones estimated using the Quantile-g computation method. (A1) (B1) (C1) (D1) (E1) The weighs represent the proportion of positive or negative components of each BPs contributing to TSH, T3, T4, FT3 and FT4. (A2) (B2) (C2) (D2) (E2) The overall joint association are interpreted as the changes of thyroid hormones level for each quantile increase in the mixture index.

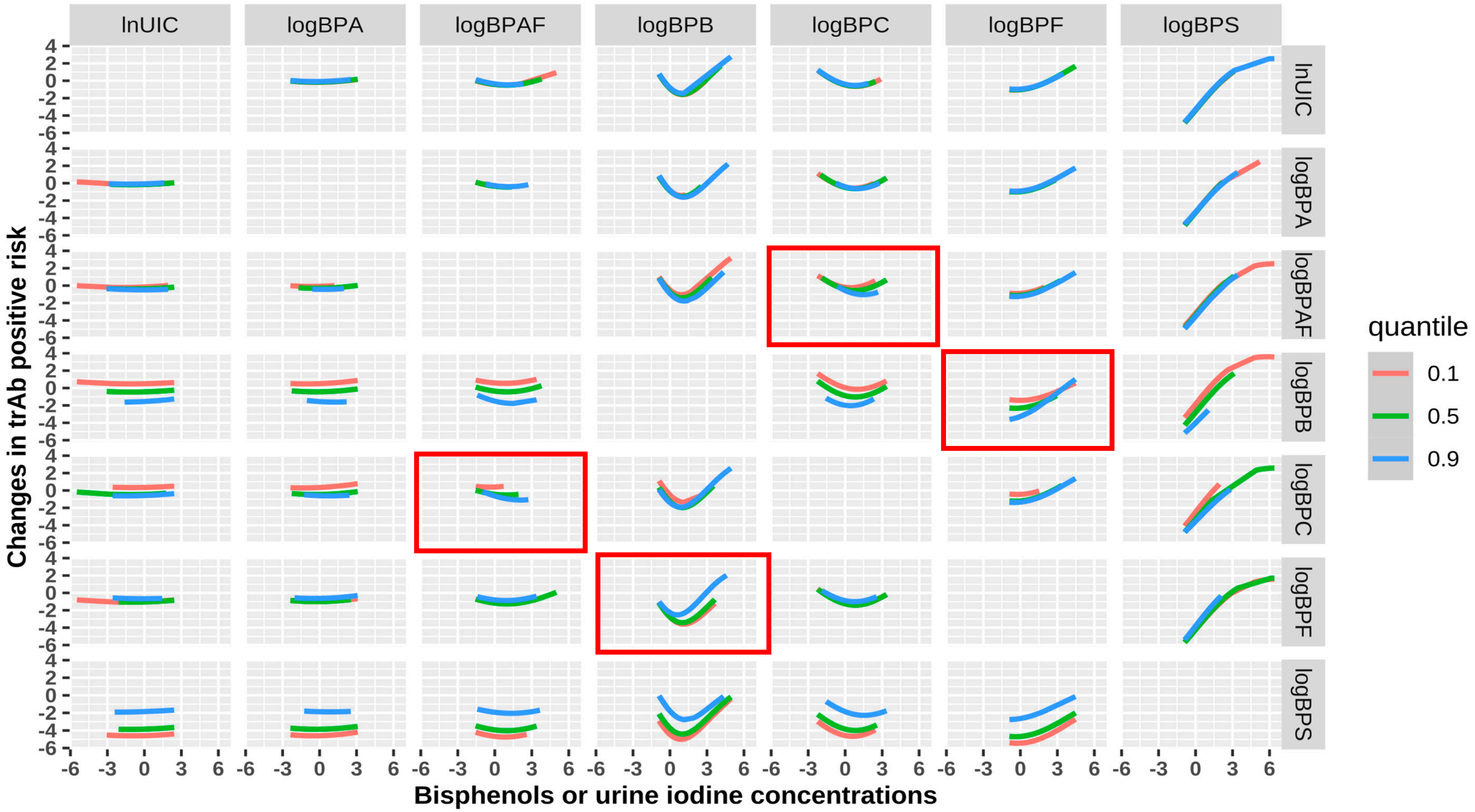

Figure S7. Interaction between pairs of exposures on TrAb positive risk

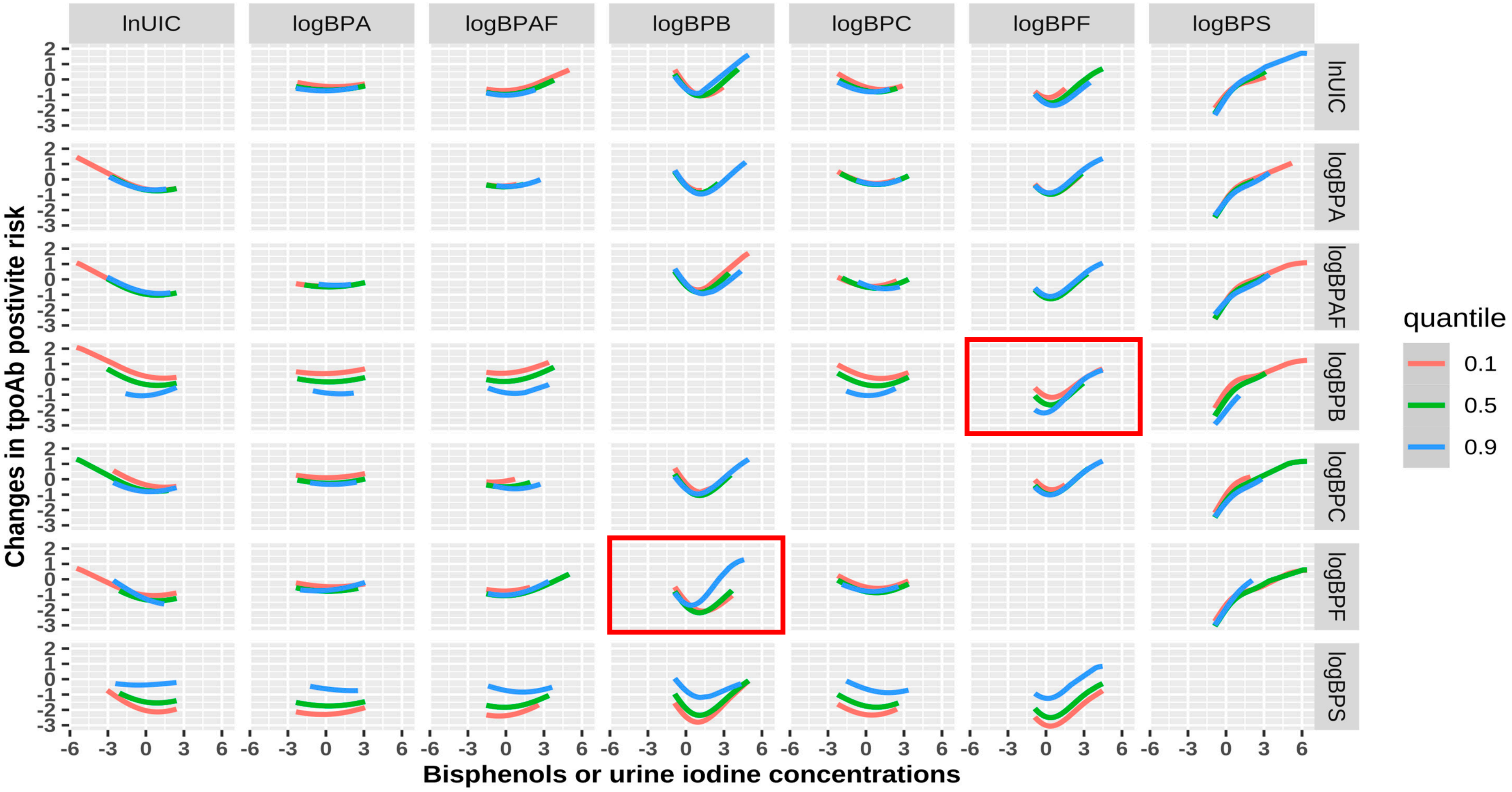

Figure S8. Interaction between pairs of exposures on TpoAb positive risk

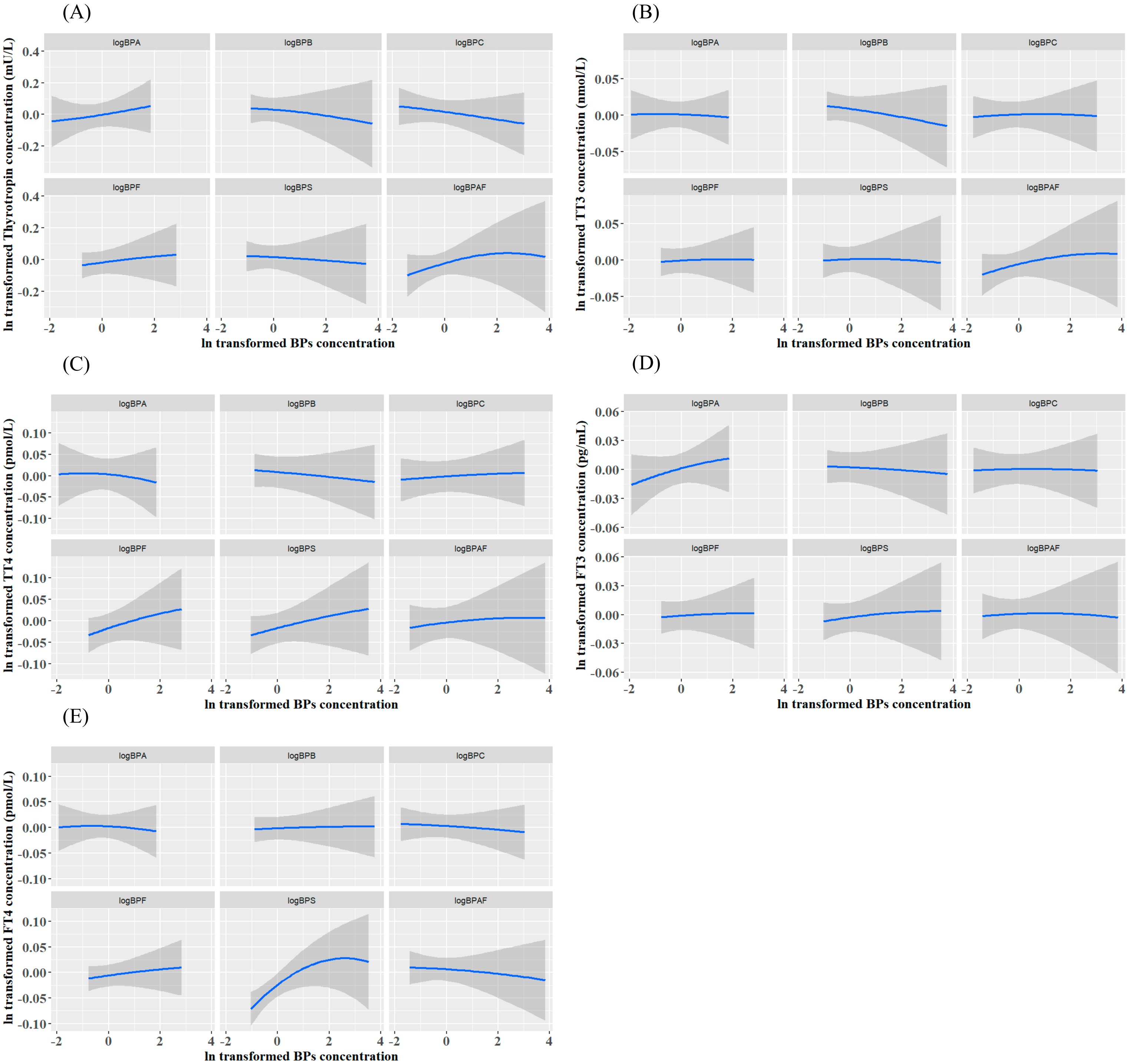

Figure S9. Univariate exposure–response functions and 95% credible intervals for each ln-transformed bisphenol (BPA, BPB, BPC, BPF, BPS, BPAF) and thyroid hormones in women with normal UIC. (A) (B) (C) (D) (E) Univariate exposure–response functions between each bisphenol exposure and serum thyrotropin, TT3, TT4, FT3 or FT4.

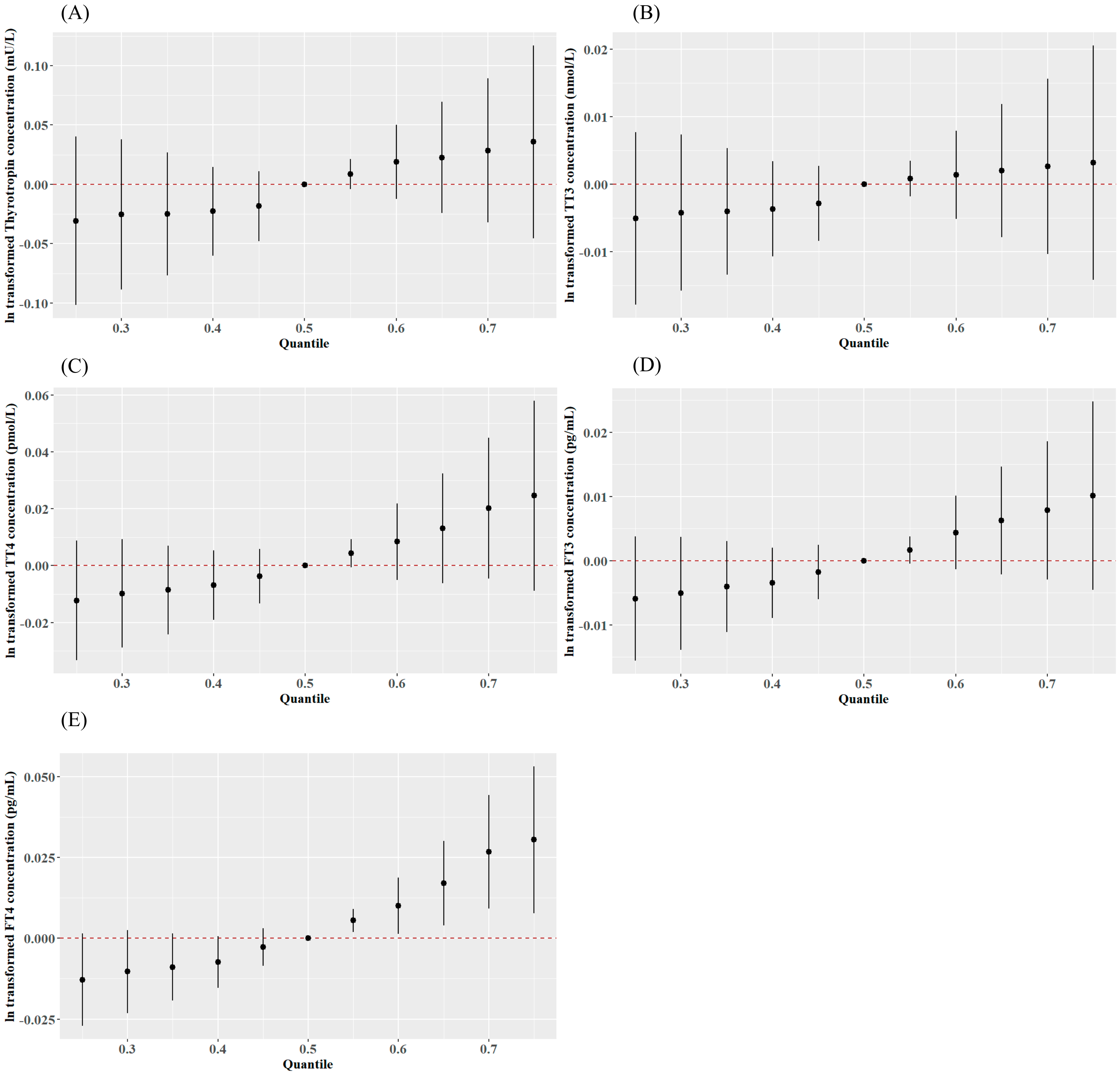

Figure S10. Combined effects of BPs mixture on thyroid hormones in women with normal UIC. (A) (B) (C) (D) (E) Overall effects of BPs mixture on thyrotropin, TT3, TT4, FT3 or FT4.

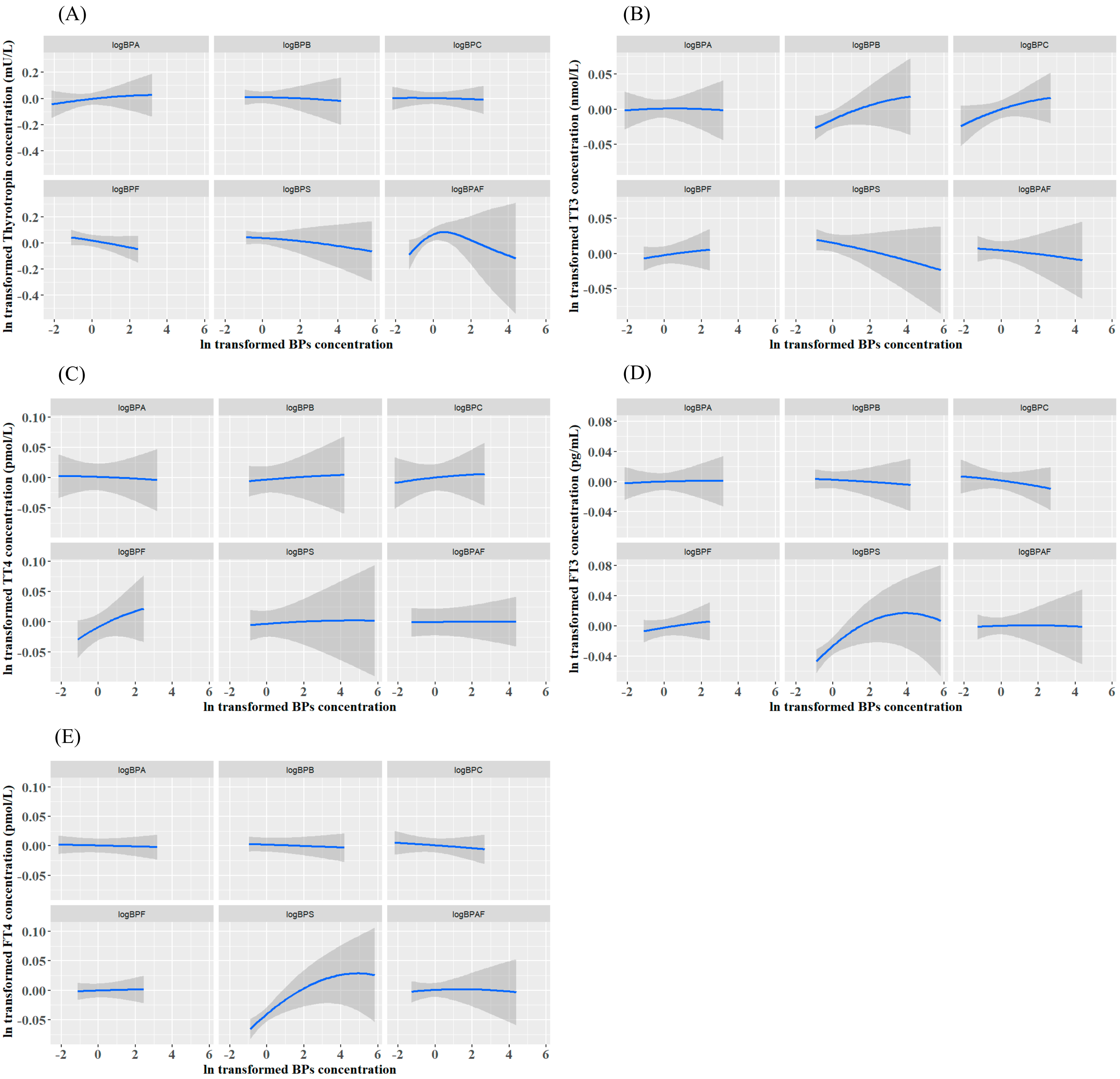

Figure S11. Univariate exposure–response functions and 95% credible intervals for each ln-transformed bisphenol (BPA, BPB, BPC, BPF, BPS, BPAF) and thyroid hormones in women with low UIC. (A) (B) (C) (D) (E) Univariate exposure–response functions between each bisphenol exposure and serum thyrotropin, TT3, TT4, FT3 or FT4

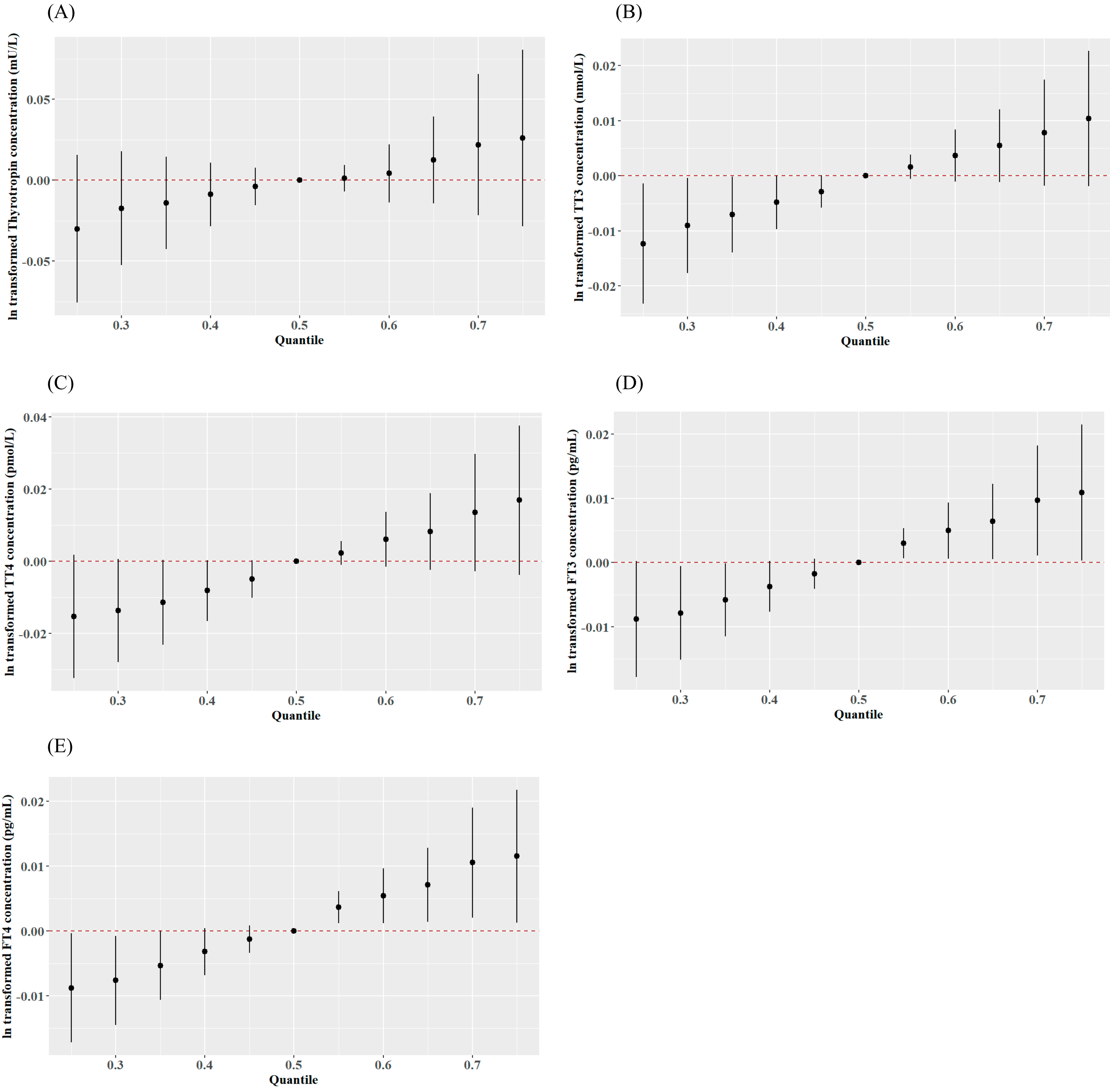

Figure S12. Combined effects of BPs mixture on thyroid hormones in women with low UIC. (A) (B) (C) (D) (E) Overall effects of BPs mixture on thyrotropin, TT3, TT4, FT3 or FT4.

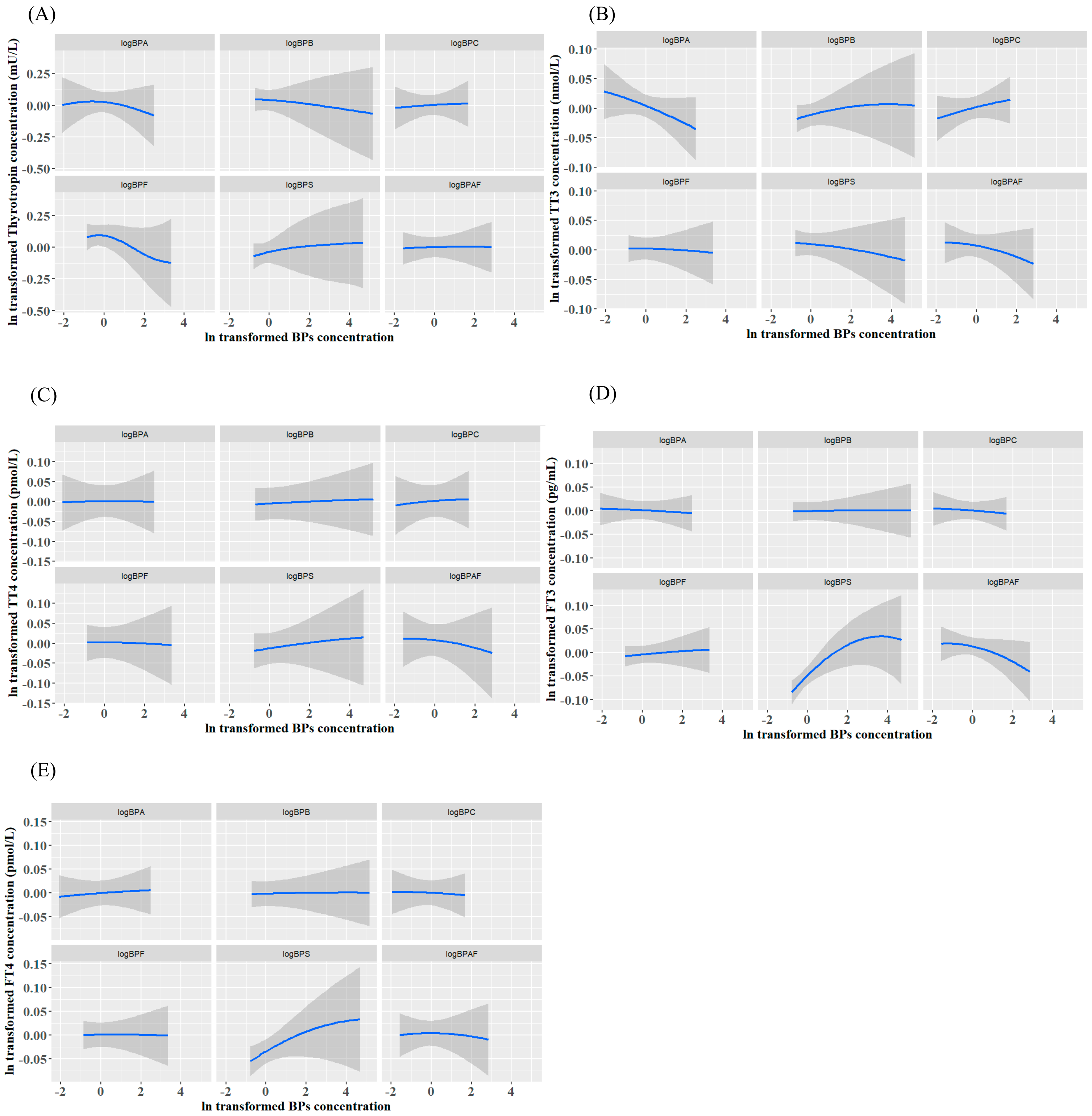

Figure S13. Univariate exposure–response functions and 95% credible intervals for each ln-transformed bisphenol (BPA, BPB, BPC, BPF, BPS, BPAF) and thyroid hormones in women with high UIC. (A) (B) (C) (D) (E) Univariate exposure–response functions between each bisphenol exposure and serum thyrotropin, TT3, TT4, FT3 or FT4

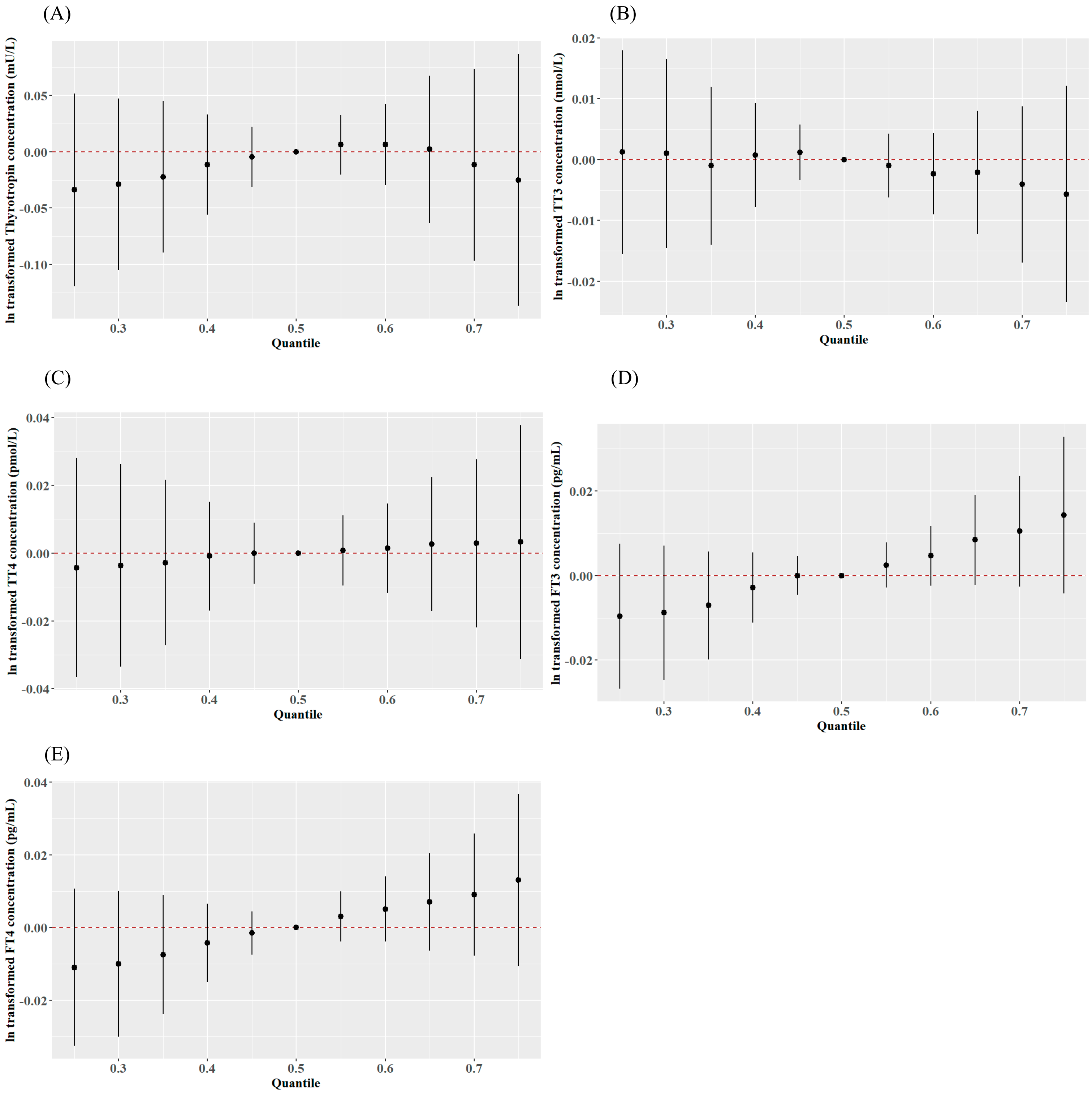

Figure S14. Combined effects of BPs mixture on thyroid hormones in women with high UIC. (A) (B) (C) (D) (E) Overall effects of BPs mixture on thyrotropin, TT3, TT4, FT3 or FT4.

(A) (B)

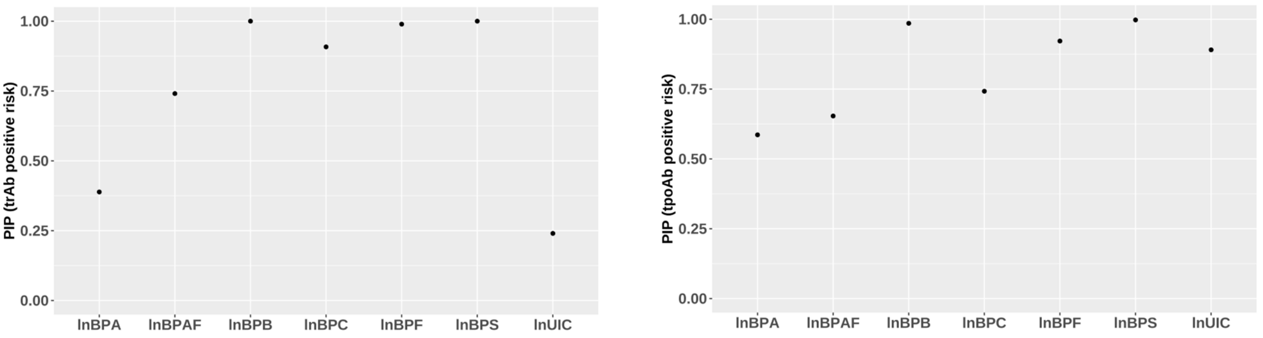

Figure S15. The posterior inclusion probabilities (PIPs) of BPs or iodine, which provide a measure of variable importance for each BP or iodine. (A) PIPs of TrAb positive risk (B) PIPs of TpoAb positive risk
